# Impact of Ablation Energy Sources on Perceived Quality of Life and Symptom in Atrial Fibrillation Patients: A Comparative Study

**DOI:** 10.3390/jcm14082741

**Published:** 2025-04-16

**Authors:** Andrea Matteucci, Maurizio Russo, Marco Galeazzi, Claudio Pandozi, Michela Bonanni, Marco Valerio Mariani, Nicola Pierucci, Vincenzo Mirco La Fazia, Stefania Angela Di Fusco, Federico Nardi, Furio Colivicchi

**Affiliations:** 1 Clinical and Rehabilitation Cardiology Division, San Filippo Neri Hospital, 00135 Rome, Italy; 2 Department of Experimental Medicine, Tor Vergata University, 00133 Rome, Italy; 3 Department of Cardiovascular, Respiratory, Nephrological, Anesthesiological and Geriatric Sciences “Sapienza” University of Rome, 00185 Rome, Italy; 4 Santo Spirito Hospital, Casale Monferrato, 15033 Alessandria, Italy

**Keywords:** atrial fibrillation ablation, quality of life, general anesthesia, pulsed-field ablation, radiofrequency

## Abstract

**Background**: Catheter ablation is a first-line treatment for rhythm control strategies in patients with atrial fibrillation (AF), with different energy sources available, including pulsed-field ablation (PFA), high-power short-duration radiofrequency (HPSD RF), conventional radiofrequency (RF), and cryoballoon ablation. Limited evidence exists on how different ablation techniques affect patient-reported outcomes, such as patients’ quality of life (QoL) and perceived symptoms. This study aims to assess the impact of ablation energy sources on reported QoL and symptom perception after AF ablation. **Methods**: The study included 148 patients who underwent catheter ablation in different centers. Patients were divided into four groups according to the energy source used. Follow-up was conducted during the 6 months post-procedure. Patients were asked to complete a 20-item questionnaire evaluating quality of life, activity resumption, recovery process, perceived symptoms, and satisfaction. Comparative analyses were performed across energy groups, anesthesia types, and anesthetic drugs. **Results**: PFA patients reported the highest improvement in QoL scores compared to RF, HPSD RF, and cryoablation (*p* < 0.001). Activity resumption and symptom relief were significantly better in the PFA group compared to others (*p* < 0.001). Anesthesia type and anesthetic drug influenced QoL outcomes, with patients under general anesthesia showing higher QoL scores compared to deep sedation (*p* < 0.001). The energy source and anesthetic drug resulted in independent predictors of QoL improvement. **Conclusions**: Ablation energy source could impact patients’ perceived QoL and symptom relief after AF ablation. PFA demonstrated superior performance scores in QoL and symptom perception compared to other techniques. Anesthetic drugs also play a role in patient-reported outcomes and activity resumption.

## 1. Introduction

Atrial fibrillation (AF) is the most frequently diagnosed arrhythmia in clinical practice, particularly among the elderly population. Its prevalence is expected to rise globally due to the aging population and increased survival rates in cardiovascular disease patients [1]. AF poses a significant public health challenge, as it is closely associated with increased morbidity and mortality, mainly driven by heart failure (HF) and thromboembolic complications, particularly stroke. The clinical burden of AF extends beyond these life-threatening events, profoundly affecting patients’ quality of life (QoL) due to symptoms such as palpitations, fatigue, dyspnea, and psychological distress [2].

Current treatment strategies for AF aim to alleviate symptoms, prevent complications, and improve QoL. Rhythm control strategies, which seek to restore and maintain sinus rhythm, have gained increasing attention in recent years, as accumulating evidence suggests that rhythm control provides superior symptom relief and QoL outcomes compared to rate control strategies [3,4]. Among the available rhythm control options, catheter ablation has emerged as a cornerstone therapy, offering higher efficacy than antiarrhythmic drugs (AADs) both as first-line treatment and after AAD failure or intolerance [5].

Several energy sources are currently employed for AF ablation, including radiofrequency (RF), cryoballoon ablation, high-power short-duration radiofrequency (HPSD RF), and novel pulsed field ablation (PFA). Each technique presents distinct biophysical properties, procedural characteristics, and potential clinical outcomes. While numerous studies have focused on the efficacy and safety of different ablation techniques in terms of arrhythmia recurrence, less attention has been devoted to their impact on patient-reported outcomes, such as QoL and perceived symptom relief [6,7].

Understanding how different ablation energy sources affect these outcomes could help in optimizing patient care and personalizing treatment approaches. Our study aims to assess the impact on QoL and symptom perception after AF ablation by comparing different energy sources.

## 2. Materials and Methods

### 2.1. Study Design and Population

We conducted a multicenter observational study involving 148 patients who underwent AF ablation between January 2022 and November 2024. Eligible patients were aged between 18 and 80 years with a diagnosis of paroxysmal or persistent symptomatic AF. Patients eligible for catheter ablation included those who were symptomatic for AF or intolerant to AADs with an indication to ablation strategy, with or without previous ablation procedures, according to the latest recommendations [3,4]. Among the study population, a small number of patients had a history of previous electrophysiological procedures. These included typical atrial flutter ablation, atrioventricular nodal reentrant tachycardia (AVNRT) ablation, and electrophysiological studies. No patient had undergone prior pulmonary vein isolation. Exclusion criteria were severe structural heart disease and the inability to provide informed consent. All patients provided written informed consent before undergoing the ablation procedure. The study was conducted in accordance with the principles of the Declaration of Helsinki. All procedures performed were part of routine clinical practice without additional interventions.

Patients underwent catheter ablation using one of four different techniques and technologies available: PFA with non-thermal delivery of electric fields to induce selective cardiomyocyte necrosis, conventional RF, HPSD RF, aiming to create rapid lesions with minimal collateral damage, or cryoballoon ablation, using freezing temperatures to achieve pulmonary vein isolation.

The choice of energy modality was based on operator preference, the availability of the method in the centers, and patient characteristics. Previous procedures did not influence the choice of energy source or anesthesia strategy and were not considered to affect the quality of life outcomes evaluated in this study. Transseptal puncture was performed under radioscopic guidance or intracardiac echocardiography, using either pressure or radiofrequency needles.

### 2.2. Anesthesia

Anesthesia protocols included either general anesthesia with orotracheal intubation or deep sedation with a laryngeal mask airway, based on patient characteristics, choice of anesthesiologist, and center protocols. General anesthesia was performed to ensure complete immobility and optimal patient comfort during the procedure, particularly in cases of prolonged or complex ablations. It involved the administration of propofol, a short-acting sedative–hypnotic agent, often combined with an ultra-short-acting opioid analgesic, to provide both sedation and analgesia. Orotracheal intubation was used to secure the airway and enable mechanical ventilation, ensuring adequate oxygenation and ventilation throughout the procedure. Deep sedation, selected in cases where shorter or less complex procedures were anticipated, was performed to maintain spontaneous breathing. Airway management was achieved using a laryngeal mask airway, allowing for airway patency while avoiding the need for endotracheal intubation. Hemodynamic and respiratory parameters were continuously monitored in both anesthesia protocols to ensure patient safety. The choice of anesthesia type and specific drug regimen was recorded for subsequent subgroup analyses to assess their potential influence on patient-reported outcomes.

### 2.3. Follow-Up

Patients underwent follow-up at 1 month and 6 months post-procedure to assess clinical outcomes and patient-reported perceptions. The 1-month follow-up visit included a comprehensive clinical evaluation to document any recurrence of AF symptoms, identify late complications, and adjust pharmacological therapy when necessary. This visit aimed to assess procedural efficacy early and optimize ongoing treatment strategies. At the 6-month follow-up, patients were invited to complete a structured 20-item questionnaire to evaluate their perception of the ablation’s impact on their quality of life and symptom burden. The questionnaire encompassed five key domains: quality of life, activity resumption, recovery process, perceived symptoms, and satisfaction with the procedure. Each domain included four targeted questions, exploring different aspects of patient well-being and functional recovery. Responses were rated on a 1-to-5 Likert scale, where higher scores reflected greater perceived improvement. The total score was obtained by summing all 20 item scores, yielding a comprehensive evaluation ranging from 20 to 100 points (see Table 1).

## 3. Statistical Analysis

Descriptive statistics are presented as means ± SD for normally distributed continuous variables or as medians with IQR for non-normally distributed data. Categorical variables are presented as absolute numbers (percentages). The normality of distribution was assessed using the nonparametric Kolmogorov–Smirnov test. Differences between means were assessed using the *t*-test for normally distributed variables, with the F-test used to assess the equality of variances. For non-normally distributed variables, the nonparametric Mann–Whitney test was employed. Proportional differences were evaluated using either χ2 analysis or Fisher’s exact test as appropriate. The analysis of variance (ANOVA), followed by Dunnett’s two-sided post hoc test for multiple comparisons, was used. Linear regression models were applied to identify independent predictors of QoL improvement. A significance threshold of *p* < 0.05 was applied to all tests. Statistical analyses were conducted with STATA statistical software, V.16.0 (College Station, TX, USA: StataCorp) and R Studio (version 2024.12.1, Posit Software, Boston, MA, USA).

## 4. Results

A total of 148 patients who underwent atrial fibrillation (AF) ablation were included in this study. Patients were stratified into four groups based on the energy source used: pulsed-field ablation (PFA, *n* = 52), high-power short-duration radiofrequency (HPSD RF, *n* = 45), conventional radiofrequency (RF, *n* = 42), and cryoballoon ablation (Cryo, *n* = 10). The mean age of the overall cohort was 63.4 ± 7.9 years. The majority of patients were male (68%). The characteristics of the study population are detailed in Table 2.

Regarding comorbidities, 32.3% of patients had diabetes, 52.0% had dyslipidemia, and 57.7% had hypertension. Although there was a trend toward higher diabetes prevalence in the RF group (43.5%) compared to other groups, this difference did not reach statistical significance. Hypertension and dyslipidemia were similarly distributed among groups, and 77% of patients presented paroxysmal AF, while 23% presented persistent AF. Patients with persistent AF were clinically stable, without significant left atrial enlargement or structural heart disease. When comparing outcomes between the two groups, no statistically significant differences were observed in terms of acute procedural success, complication rates, or arrhythmia recurrence, or the scores achieved.

### 4.1. Quality of Life and Symptom Improvement

**Figure 1:**
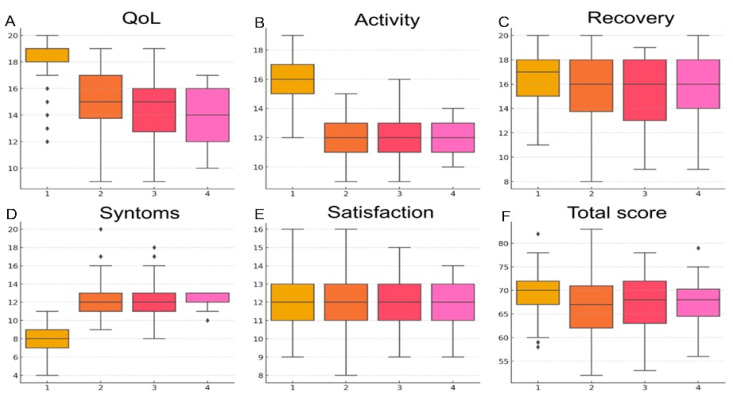
Boxplots illustrating patient-reported outcomes stratified by ablation energy source. The graphs display comparisons of (**A**) quality of life (QoL), (**B**) activity resumption, (**C**) post-ablation recovery, (**D**) symptom perception, (**E**) satisfaction, and (**F**) total score across four energy types: 1 = pulsed-field ablation (PFA), 2 = high-power short-duration radiofrequency (HPSD RF), 3 = conventional radiofrequency (RF), and 4 = cryoballoon ablation.

Significant differences were observed in patient-reported outcomes based on the ablation energy source used (Figure 1).

Patients undergoing PFA exhibited the greatest improvement in quality of life (QoL) scores (18.2 ± 1.7) compared to those treated with HPSD RF (15 ± 2.3), RF (14.5 ± 2.7), and Cryo (13.7 ± 2.5) (*p* < 0.001). Similarly, activity resumption scores were highest in the PFA group (15.9 ± 1.8), significantly exceeding those of HPSD (12.0 ± 1.4), RF (11.9 ± 1.6), and Cryo (12.1 ± 1.2) (*p* < 0.001). Patients treated with PFA also reported the most significant relief from symptoms (7.9 ± 1.6), whereas symptom scores were markedly higher in the HPSD (12.3 ± 2), RF (12.2 ± 2.5), and Cryo (12.2 ± 1.1) groups (*p* < 0.001). Overall satisfaction scores were similar across groups, with no significant differences (*p* = 0.94). The total composite score, encompassing all questionnaire domains, was significantly higher in the PFA group (69.7 ± 4.5) compared to HPSD (66.7 ± 6.1), RF (67.1 ± 6), and Cryo (67.4 ± 5.6) (*p* = 0.001).

### 4.2. Influence of Anesthesia and Anesthetic Drugs

Anesthesia type and anesthetic drug choice significantly influenced patient-reported outcomes. General anesthesia was administered to 75% of patients, while 25% underwent deep sedation. Patients who received general anesthesia exhibited significantly better QoL scores (17.1 ± 2.0) compared to those under deep sedation (12.5 ± 2.5) (*p* < 0.001). Activity resumption and post-ablation recovery scores were also significantly higher in the general anesthesia group (*p* < 0.001), while symptom burden and satisfaction scores remained comparable between groups. The total composite score was significantly higher in patients receiving general anesthesia (70.2 ± 3.9) than those under deep sedation (60.9 ± 4.3) (*p* < 0.001) (Table 3).

**Figure 2:**
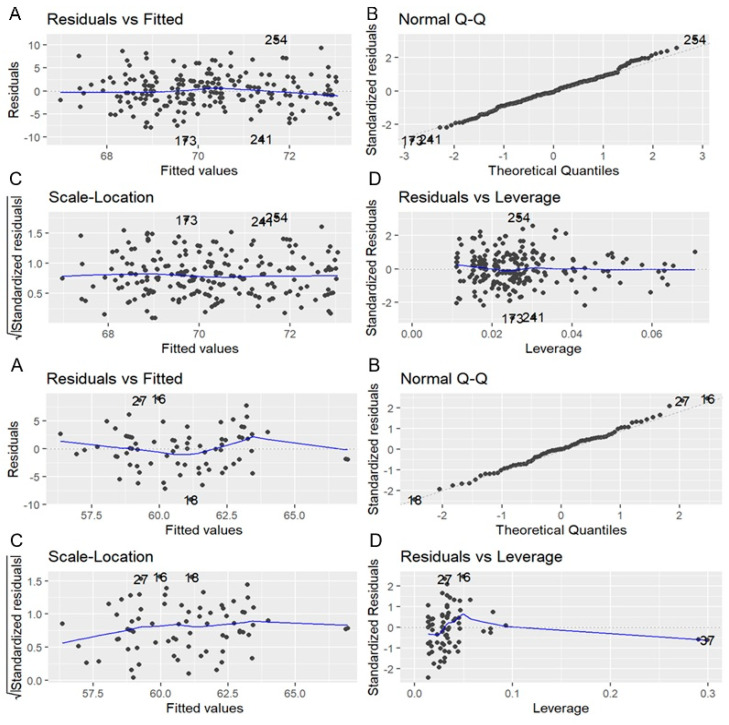
Diagnostic plots for linear regression model focused on patients undergoing atrial fibrillation ablation under general anesthesia with propofol and remifentanil. The plots include the following: (**A**) Residuals vs. Fitted values, (**B**) Normal Q–Q plot, (**C**) Scale–Location plot, and (**D**) Residuals vs. Leverage plot. The residuals show an approximately random scatter, suggesting linearity and homoscedasticity assumptions are met. The Q–Q plot indicates normal distribution of residuals. No influential outliers are evident based on leverage values.

Among patients under general anesthesia, those receiving remifentanil demonstrated superior total scores (71.7 ± 3.7) compared to those given propofol (69.3 ± 3.7) (*p* < 0.001). Post-ablation recovery was significantly better in the remifentanil group (18.3 ± 0.9) compared to the propofol group (16.1 ± 1.7) (*p* < 0.001), while other QoL parameters were comparable. Multivariate linear regression analysis identified energy source and anesthetic drug as independent predictors of QoL improvement. In patients under deep sedation, BMI (*p* = 0.041) and energy source (*p* < 0.001) significantly influenced total scores, with lower BMI and PFA predicting better outcomes. Among patients under general anesthesia, the anesthetic drug (*p* < 0.001) and energy source (*p* = 0.002) remained significant predictors, with remifentanil and PFA associated with higher scores (Table 4) (Figure 2).

## 5. Discussion

Catheter ablation is a well-established treatment for AF, with numerous studies demonstrating its efficacy in restoring sinus rhythm and reducing AF burden. However, while procedural success is often measured in terms of recurrence rates and electrophysiological endpoints, patient-centered outcomes, including QoL, symptom relief, and post-procedural recovery, are equally critical but less frequently examined. This study provides for the first time an overview of how different ablation energy sources and anesthesia strategies impact patient-reported outcomes, emphasizing the importance of considering patient experience alongside clinical efficacy. Patient-centered outcomes in AF significantly impact patients’ QoL, not only due to its symptoms but also because of the psychological burden and lifestyle limitations it imposes [7,8,9,10]. Several studies have demonstrated that ablation provides superior QoL outcomes compared to antiarrhythmic drug therapy, with sustained benefits over time [5,6]. However, while procedural success is often measured by arrhythmia recurrence, patient-centered outcomes such as symptom relief and post-procedural recovery require equal consideration.

Our study showed that the choice of ablation energy source and anesthesia could significantly influence patient-reported outcomes. Among the evaluated energy sources, PFA resulted in the greatest improvements in QoL and activity resumption, aligning with prior research suggesting that its non-thermal mechanism minimizes collateral tissue damage and facilitates a smoother recovery [11,12]. Traditional RF, particularly in its conventional form, has been associated with a higher risk of esophageal injury, phrenic nerve palsy, and post-ablation atrial stunning, potentially prolonging recovery [11,13,14,15]. Cryoballoon ablation, while effective, has been linked to transient phrenic nerve injury, which may explain its comparatively lower QoL scores [7]. These findings highlight the importance of incorporating patient-reported outcomes when selecting an ablation strategy. While previous studies have emphasized procedural success rates, our data suggest that patient perception of recovery should also guide clinical decision-making. Physicians should discuss with patients not only the expected success of each technique but also its anticipated recovery timeline and impact on daily life [16,17]. Also, our study found that patients undergoing general anesthesia reported significantly higher QoL scores compared to those receiving deep sedation. This supports existing evidence indicating that general anesthesia may improve procedural comfort, minimize intraoperative stress, and enhance post-procedural recovery by reducing patient anxiety and discomfort [18,19].

Additionally, the specific anesthetic agent used was found to influence patient-reported outcomes. Patients who received remifentanil exhibited better QoL scores and faster activity resumption compared to those given propofol. Remifentanil’s rapid metabolism and short half-life allow for a smoother recovery, with less residual sedation and fatigue [10]. In contrast, propofol, despite its widespread use, can result in prolonged sedation effects, grogginess, and post-procedural nausea, negatively affecting the patient’s perception of recovery [20]. While general anesthesia is typically reserved for complex ablation procedures requiring precise catheter control, our results suggest that even in standard cases, it may offer benefits in terms of QoL and recovery speed [9].

A crucial aspect of patient-centered care in AF ablation is the ability to return to normal daily activities. Our results show that PFA patients had the fastest recovery and highest activity resumption scores, reinforcing the idea that reducing procedural invasiveness enhances post-procedural well-being [8,20]. Furthermore, patients under general anesthesia demonstrated better activity resumption than those under deep sedation, supporting the hypothesis that a more controlled perioperative experience with lower intraoperative stress translates to improved functional recovery [21,22]. This may be related to improved catheter stability, more consistent lesion formation, and greater intra-procedural comfort, all of which may contribute to better procedural efficacy and patient experience. Based on our results, the patient profile most likely to benefit from this approach includes individuals with symptomatic paroxysmal or early persistent atrial fibrillation, without advanced structural heart disease, and those with low tolerance to conscious sedation. Previous studies have suggested that general anesthesia or deep sedation during AF ablation may be associated with lower recurrence rates compared to conscious sedation, although the difference was not statistically significant [23]. General anesthesia may contribute to improved procedural outcomes by enhancing patient immobility during the procedure, potentially improving ablation accuracy and reducing recurrence risk. This could result in better post-procedural QoL by reducing the need for further interventions and improving symptoms associated with AF. Some studies have reported a higher likelihood of blood pressure drop during deep sedation or general anesthesia, which can affect hemodynamic stability, particularly in patients with pre-existing cardiovascular conditions [24]. However, the impact on long-term outcomes remains less clear. The use of PFA under general anesthesia could potentially lead to a more significant and sustained symptomatic relief, fewer arrhythmia-related emergency department visits, and reduced overall healthcare resource consumption. By integrating patient-reported outcome measures (PROMs) into routine clinical practice, physicians can better align treatment strategies with patient expectations [13,14,25,26]. In line with current clinical practice guidelines, all patients in our study were maintained on oral anticoagulation following the procedure. However, antiarrhythmic drug (AAD) therapy was discontinued after ablation, as patients undergoing ablation are typically those who are either intolerant to AADs or remain symptomatic despite pharmacological therapy. For patients with paroxysmal or early persistent AF, maintaining AADs post-procedure does not provide additional benefit, as ablation is expected to significantly reduce arrhythmic burden. It is also important to minimize the number of medications prescribed [27], as this helps improve patient compliance, with many patients expecting to reduce their medication regimen following successful ablation.

The low recurrence rate of atrial fibrillation observed at 6 months suggests that AAD therapy did not have a significant impact on clinical outcomes in our cohort. In our study, the assessment of AF symptoms and recurrences was primarily based on patient-reported outcomes, supported by standard ECG and 24-h Holter monitoring during follow-up. At the 1-month follow-up, all patients were in sinus rhythm. At 6 months, atrial fibrillation recurrence was documented in eight patients. However, a systematic evaluation of AF burden and PAC burden through extended rhythm monitoring (e.g., long-term Holter or implantable loop recorders) was not performed across the entire cohort. As such, we were unable to establish a direct correlation between the objective arrhythmia burden and the magnitude of QoL improvement, particularly in the PFA group and in patients treated under general anesthesia. This could represent a potential area for future investigation.

Our study presents several limitations that should be acknowledged. First, the sample size, although adequate to detect meaningful trends, remains relatively modest, potentially limiting the generalizability of our findings. The subgroup of patients who underwent cryoballoon ablation was markedly smaller compared to the other energy modalities, which may have introduced imbalance and reduced the statistical power to draw firm conclusions regarding this technique. The choice of ablation strategy was not randomized, introducing possible selection bias. Lastly, the follow-up period, limited to six months, may not fully capture long-term procedural efficacy and durability of symptom improvement. Further research should explore long-term QoL trends, the psychological benefits of optimized ablation techniques, and the role of anesthesia in enhancing post-procedural recovery.

## 6. Conclusions

Our study reinforces the importance of patient-centered care in AF ablation. The choice of ablation energy source and anesthesia strategy could influence QoL, symptom relief, and post-procedural recovery. Moving beyond procedural efficacy to consider patient experience will enhance long-term satisfaction and optimize treatment outcomes. Future research should continue integrating PROMs to refine AF treatment strategies and ensure they align with patient expectations.

## Figures and Tables

**Table 1:** The 20-item questionnaire designed to evaluate the perception of the ablation’s impact on the patient’s quality of life and symptoms.

Category	Questions
Quality of Life	How would you rate the overall improvement in your quality of life?
	How much has your physical activity improved since the procedure?
	How would you rate your mental well-being after the ablation?
	How much has the procedure impacted your social life?
Resumption of Daily Activities	How easy was it to resume your usual daily activities post-ablation?
	How much has the ablation improved your ability to perform household tasks?
	How would you rate your return to work or professional activities?
	How well can you tolerate physical exertion compared to before the procedure?
Recovery After the Procedure	How would you describe your recovery process after the ablation?
	How would you rate the recovery experience following anesthesia?
	How much pain or discomfort did you experience in the days following the procedure?
	How satisfied are you with the information provided regarding post-procedure care?
Symptoms and Recurrences	How relieved do you feel from atrial fibrillation symptoms after the ablation?
	How much do you notice a reduction in the frequency of AF episodes?
	How confident are you in the procedure’s ability to prevent recurrences?
	How much have palpitations or irregular heartbeats decreased after the procedure?
Satisfaction	How satisfied are you with the ablation procedure overall?
	How confident are you in the long-term benefits of the procedure?
	How would you rate the communication and support from the medical team?
	How well has the procedure met your expectations for symptom relief?

**Table 2:** General characteristics and questionnaire scores.

	Total	PFA	HPSD RF	RF	Cryo	*p* Value
*Characteristics*	N = 148	N = 52	N = 45	N = 42	N = 10	
Age	63.4 (7.9)	63.9 (7.8)	62.5 (8.3)	63.2 (7.5)	64.5 (7.7)	0.57
Gender (Male)	100 (68%)	37 (71%)	30 (66%)	28 (68%)	6 (60%)	0.53
BMI	28.4 (3.7)	28.4 (3.8)	28.1 (3.6)	28.5 (3.7)	28.8 (4.3)	0.83
Diabetes	49 (32%)	14 (27%)	13 (30%)	18 (43%)	2 (20%)	0.15
Dyslipidemia	82 (55%)	30 (56%)	20 (45%)	23 (55%)	4 (40%)	0.39
Hypertension	92 (62%)	28 (53%)	25 (56%)	26 (63%)	6 (60%)	0.46
Anesthesia	111 (75%)	40 (77%)	33 (73%)	31 (72%)	7 (70%)	0.65
AF						0.34
Parossistic	115 (77%)	41 (78%)	33 (73%)	35 (83%)	6 (60%)	
Persistant	33 (23%)	11 (21%)	12 (26%)	7 (16%)	3 (30%)	
*Questionnaire*						
Activity	13.4 (2.4)	15.9 (1.8)	12 (1.4)	11.9 (1.6)	12.1 (1.2)	<0.001
Recovery	15.8 (2.8)	16.3 (2.3)	15.5 (3.1)	15.4 (2.8)	15.5 (3.4)	0.39
QoL	16.1 (2.8)	18.2 (1.7)	15 (2.3)	14.5 (2.7)	13.7 (2.5)	<0.001
Satisfaction	11.9 (1.7)	11.9 (1.8)	11.9 (1.79)	12 (1.6)	11.6 (1.7)	0.94
Syntoms	10.7 (2.9)	7.8 (1.6)	12.3 (2)	12.2 (2.5)	12.2 (1)	<0.001
Total Score	67.9 (5.6)	69.7 (4.5)	66.7 (6.1)	67.1 (6)	67.4 (5.6)	0.001

PFA = pulsed-field ablation, HPSD RF = high-power short-duration radiofrequency, RF = conventional radiofrequency, Cryo = cryoballoon ablation, BMI = body mass index, AF = atrial fibrillation, QoL = quality of life.

**Table 3:** Score among different anesthesia protocols and drugs.

	Total	Deep Sedation	General Anesthesia	*p* Value	Propofol	Remifentanil	*p* Value
	N = 148	N = 37	N = 111		N = 69	N = 42	
Activity	13.4 (2.5)	13.2(2.5)	13.4 (2.5)	0.64	13.4 (2.4)	13.5 (2.6)	0.83
Recovery	15.8 (2.8)	11.8 (1.5)	17 (1.7)	<0.001	16.1 (1.7)	18.3 (.9)	<0.001
QoL	16 (2.8)	12.5 (2.5)	17.1 (2)	<0.001	17 (1.8)	17 (2.1)	0.90
Satisfaction	11.9 (1.7)	11.8 (1.5)	11.9 (1.8)	0.79	11.8 (1.9)	12.1 (1.6)	0.38
Syntoms	10.7 (2.9)	10.7 (1.8)	10.7 (3.1)	0.93	10.6 (2.9)	10.7 (3.5)	0.87
Total Score	67.9 (5.6)	60.8 (4.2)	70.2 (3.9)	<0.001	69.3 (3.7)	71.7 (3.7)	<0.001

QoL = quality of life.

**Table 4:** Linear regression in different anesthesia protocols.

	Coeffient	Standard Error	*p* Value	Confidence Interval	
*Deep Sedation*					
*Age*	−0.04	0.07	0.579	−0.173	0.0958
Gender	−0.96	1.2	0.426	−3.35	1.432
BMI	−0.27	0.13	0.041	−0.537	−0.0113
AF	−0.9	1.37	0.513	−3.64	1.836
Energy	−1.98	0.49	0.0	−2.953	1.001
*General Anesthesia*					
Age	0.02	0.03	0.535	−0.0437	0.0839
Gender	0.21	0.54	0.704	−0.859	1.271
BMI	−0.08	0.06	0.21	−0.198	0.0439
AF	−0.44	0.65	0.497	−1.719	0.837
Anesthetic Drug	2.51	0.51	0.001	1.505	3.504
Energy	−0.75	0.24	0.002	−1.226	−0.265

BMI = body mass index, AF = atrial fibrillation.

## Data Availability

Data are available under reasonable request from the corresponding author.
